# Literature-based condition-specific miRNA-mRNA target prediction

**DOI:** 10.1371/journal.pone.0174999

**Published:** 2017-03-31

**Authors:** Minsik Oh, Sungmin Rhee, Ji Hwan Moon, Heejoon Chae, Sunwon Lee, Jaewoo Kang, Sun Kim

**Affiliations:** 1 Department of Computer Science and Engineering, Seoul National University, Seoul, Republic of Korea; 2 Interdisciplinary Program in Bioinformatics, Seoul National University, Seoul, Republic of Korea; 3 Division of Computer Science, Sookmyung Women’s University, Seoul, Republic of Korea; 4 Department of Computer Science and Engineering, Korea University, Seoul, Republic of Korea; 5 Bioinformatics Institute, Seoul National University, Seoul, Republic of Korea; University of São Paulo, BRAZIL

## Abstract

miRNAs are small non-coding RNAs that regulate gene expression by binding to the 3′-UTR of genes. Many recent studies have reported that miRNAs play important biological roles by regulating specific mRNAs or genes. Many sequence-based target prediction algorithms have been developed to predict miRNA targets. However, these methods are not designed for condition-specific target predictions and produce many false positives; thus, expression-based target prediction algorithms have been developed for condition-specific target predictions. A typical strategy to utilize expression data is to leverage the negative control roles of miRNAs on genes. To control false positives, a stringent cutoff value is typically set, but in this case, these methods tend to reject many true target relationships, i.e., false negatives. To overcome these limitations, additional information should be utilized. The literature is probably the best resource that we can utilize. Recent literature mining systems compile millions of articles with experiments designed for specific biological questions, and the systems provide a function to search for specific information. To utilize the literature information, we used a literature mining system, BEST, that automatically extracts information from the literature in PubMed and that allows the user to perform searches of the literature with any English words. By integrating omics data analysis methods and BEST, we developed Context-MMIA, a miRNA-mRNA target prediction method that combines expression data analysis results and the literature information extracted based on the user-specified context. In the pathway enrichment analysis using genes included in the top 200 miRNA-targets, Context-MMIA outperformed the four existing target prediction methods that we tested. In another test on whether prediction methods can re-produce experimentally validated target relationships, Context-MMIA outperformed the four existing target prediction methods. In summary, Context-MMIA allows the user to specify a context of the experimental data to predict miRNA targets, and we believe that Context-MMIA is very useful for predicting condition-specific miRNA targets.

## Introduction

MicroRNAs (miRNAs) are small non-coding RNAs that are 19-24 nucleotides in length. These RNAs regulate gene expression at the post-transcriptional level by binding to the 3′-UTR of mRNAs [1, 2]; thus, miRNAs are functionally important. There are numerous scientific findings on the functional roles of miRNAs by regulating specific genes. For example, it is reported that miR-15 and miR-16-1 bind to BCL2 [3] and that apoptosis is induced. Another example is that miR-125b, miR-145, miR-21 and miR-155 are dysregulated in breast cancer cells, and different expression levels of these miRNAs have significant correlations with breast cancer phenotypes, such as tumor stages and status of estrogen and progesterone receptors [4]. Moreover, it is well known that miRNAs are related to proliferation, differentiation, and cell death [5].

The functional roles of miRNAs differ in different contexts. In other words, the relationship between miRNA and target genes is dynamic in different conditions. Thus, it is very important to identify which genes are targeted by miRNAs in a given context. There are more than 1000 miRNAs, and approximately 60% of protein-coding genes are regulated by miRNAs [6]. Since it is not possible to perform biological experiments for such a large number of miRNAs and genes, computational prediction is very important, and numerous computational methods have been developed for predicting targets of miRNAs. The first generation of computational tools leverage sequence complementary information and binding energy potentials. These prediction methods include TargetScan [7], PITA [8], mirSVR [9], miRanda [10] and PicTar [11]. These tools generally come with corresponding databases that compile miRNA-target information. In addition to sequence complementary information, there are different approaches used in each of these methods. miRanda estimates the energy on sequence matching of miRNA and mRNA pairs to predict targets [10]. PicTar first finds candidate 3′-UTR sites and uses a hidden Markov model (HMM) to filter out target sites [11]. TargetScan considers a conservation seed match and then considers regions outside seed matches [7]. The mirSVR algorithm uses a support vector regression method to compute scores on candidate target sites that are identified by miRanda [9]. PITA uses the accessibility of target sites as a main feature to predict targets [8].

Target prediction methods based on the sequence similarity score rely on the existence of target sites, and these methods are accompanied by target databases. However, such target information is not condition specific without considering which miRNAs and which genes are expressed; thus, there are many false positives even if the target information is accurate, which is not the case since many target databases do not agree on the miRNA-target relationship. To make the target information condition specific, many expression-based target prediction methods have been developed. These methods take miRNA-mRNA expression data and several sequence-based target databases as input data and filter out miRNA-mRNA targets using statistical significance or computational algorithms. We briefly summarize the previous expression-based algorithms. GenMiR++ used a Bayesian model and expectation maximization algorithm to predict the posterior probability of a miRNA target for mRNA [12]. MMIA employs a two-step method, where the first step is to select differentially expressed miRNA, and the second step is to select negatively correlated differentially expressed mRNA [13] only for the differentially expressed miRNAs. MMIA also supports sequence data analysis on a cloud environment, which enables the user to utilize both microarray data and NGS data [14]. MAGIA2 is a web-based tool that considers the correlation among miRNA and mRNA and transcription factor (TF) regulation [15]. CoSMic extracts the significant target mRNA cluster for each miRNA [16]. CoSMic employs methods similar to gene set enrichment analysis (GSEA) to identify miRNA targets [17]. miRNAmRNA is a target prediction algorithm based on the global test of a linear regression model [18]. To extract condition-specific miRNA activity, identifying causal relationships using intervention calculus when the DAG is absent was proposed [19]. A recent tool, PlantMirnaT, was designed as a plant-specific miRNA-mRNA sequencing data analysis algorithm [20]. The unique feature of PlantMirnaT is using the expression quantity information from sequencing data and employing a split ratio model to identify the relationship of target pairs.

## Motivation

There are approximately 1,500 known miRNAs in the human genome. The number of possible miRNA-gene pairs exceeds 30 million when more than 20,000 protein-coding genes are considered. Among these pairs, only a fraction of the relationships are significant in terms of biological functions, e.g., phenotypes or cancer subtypes. Computational methods for predicting the miRNA target employ various techniques to identify phenotype-specific miRNA targets. Because this is a typical prediction problem, the challenges can be summarized in terms of false positives and false negatives.

- **Target databases have high false positive rates**: Sequence-based target prediction algorithms, such as TargetScan, mirSVR, and PITA, and their corresponding databases generally produce high false positives. There are two major reasons for these high false positives. First, these databases contain all known targets; thus, the target information is not condition specific. For this reason, when transcriptome data measured in a specific condition are analyzed, many targets are false positives. Second, sequence-based prediction methods do not consider the regulatory role of miRNA, which generally results in a negative correlation between miRNA and the target gene. In addition, sequence-based prediction methods do not consider sample-specific sequence information. For example, sequence variations in the target regions can affect the target relationship, but the current algorithms do not consider minor but subtle sequence variations.
- **Expression-based methods may have false negative rates**: Expression-based methods utilize negative correlation information between miRNA and targets or similar approaches. For these methods, there is always an issue of establishing a cutoff threshold value, e.g., for a negative correlation. If the cutoff value is not stringent, then there are too many miRNA-target relationships. Thus, in general, it is a common practice to set a quite stringent cutoff value. In this case, many true miRNA-target relationships can be rejected, i.e., the false negative issue.

**Fig 1:**
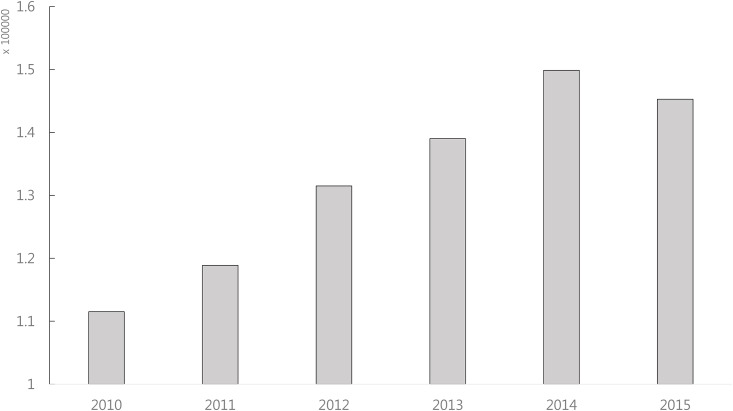
The number of published papers related to the keyword ‘cancer’ since 2010. More than 100,000 papers have been published every year.

Addressing the false positive and false negative issues is a very challenging problem unless we fully understand how miRNAs regulate target genes. Using sequence pairing information and gene expression information is very useful because such methods have already produced many biologically meaningful results. However, one important information source, the literature, is not utilized in current methods. The scientific literature is currently growing exponentially. As shown in Fig 1, more than 100,000 papers related to ‘cancer’ are published every year. Thus, if we combine sequence pairing information and gene expression information with the literature information, we can certainly make a good improvement in predicting miRNA targets, reducing both false positives and false negatives. In particular, as with the use of gene expression information, the use of the literature information should be condition specific. The main issues are how to handle the vast amount of studies in the literature, how to allow the user to specify the experimental conditions, and finally, how to combine sequence pairing information, gene expression information and the literature information in a single computational framework.

Toward this goal, two research groups are working together to design and implement a novel human-specific miRNA-target prediction method.

First, we compute the **omics score** by utilizing sequence pairing information and gene expression information to produce candidate miRNA-target pairs. Then, we compute the literature-based **context score** to evaluate each candidate miRNA-target pair using the Biomedical Entity Search Tool (BEST) [21]. Using BEST, the user can specify the experimental condition using a set of any keywords, which will automatically be translated to a set of genes and related miRNAs. Subsequently, the two scores, the **omics score** and the **context score**, are combined into a single score in a conditional probabilistic form.

The remainder of this paper is organized as follow. In the Methods section, we explain how to compute the **omics score** based on the expression data and miRNA-gene relationship and the **context score** from the literature according to user-provided keywords. In the Results section, we show how our proposed method performs compared with four existing methods in experiments with omics datasets in the public domain.

## Methods

**Fig 2:**
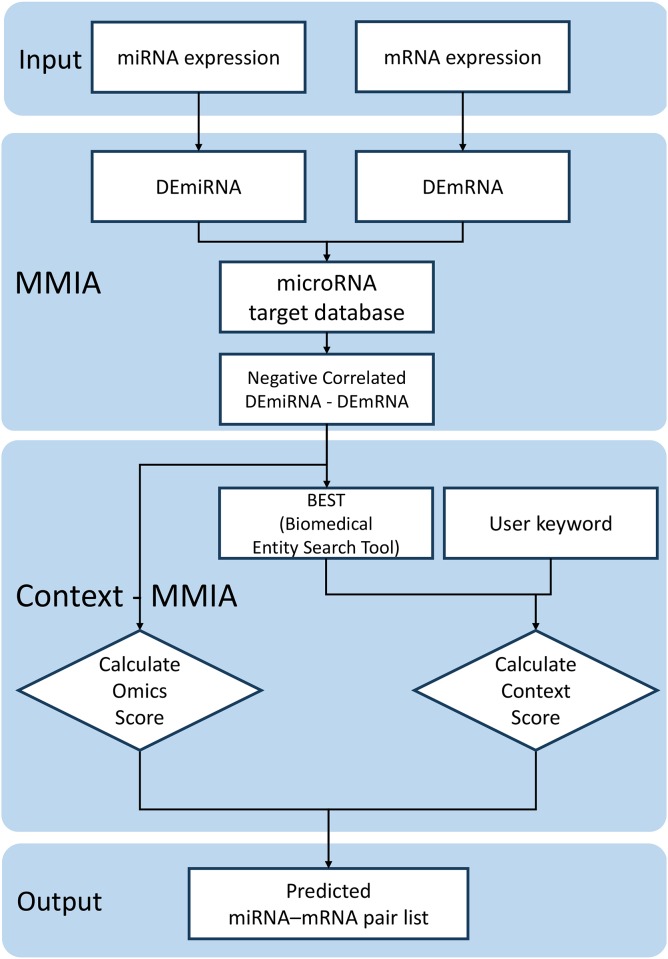
Schematic workflow for Context-MMIA. The system accepts expression information of miRNA and mRNA as inputs. In the MMIA step, DEmiRNAs and DEmRNAs are extracted based on their expression level difference, and their negative correlation is computed. In the Context-MMIA step, the system computes omics and context scores based on user-provided keywords by utilizing the BEST system. Finally, the system ranks miRNA-mRNA pairs using the scores.

In this section, we explain how our method, Context-MMIA, predicts human miRNA targets by combining the literature information and gene expression data. Context-MMIA takes two-class (control vs. treated) human miRNA-mRNA expression data as input. Then, with user-specified keywords as the context of the experiment, it computes the probabilities of miRNA-gene pairs relevant to the phenotype differences by combining gene/miRNA expression data and the literature data. Fig 2 illustrates the workflow of Context-MMIA. First, differentially expressed miRNAs (DEmiRNAs) and differentially expressed mRNAs or genes (DEmRNAs) are determined with a cutoff value at the relaxed level such that most of the true positives can be retained in this step. Note that we use negative correlation information and the literature information to filter out and re-weight candidates for interaction pairs in the following steps. In the second step of processing omics data, human miRNA-mRNA pairs are predicted using miRNA target databases such as TargetScan, mirSVR, and PITA. These miRNA-mRNA pairs are further screened by negative correlation information between miRNA and mRNA. In the third step, for each pair of miRNA and mRNA, Context-MMIA calculates the **omics score** based on expression data and the **context score** based on the literature information compiled based on the user-provided keywords. Finally, target pairs are ranked by combining the **omics score** and **context score**. For each miRNA-mRNA pair, Context-MMIA computes alignments of human miRNA and the 3′-UTR of mRNA and generates the visualization of the miRNA-mRNA alignment on the website.

### Identifying genes and miRNAs based on the user-provided context

Context-MMIA takes a set of keywords from the user to specify the context of the experiment. Currently, the most widely used biomedical literature database, PubMed, contains over 26 millions records. When we perform a search with the keyword ‘cancer’, over 3 million records are retrieved. Thus, we believe that this literature database contains enough articles to rank miRNA-gene pairs in terms of the user-provided context. However, there are two major issues in ranking miRNA-gene pairs: given the keywords, relevant papers should be identified and relevant gene names and miRNA names should also be identified. Since not all papers contain the user-provided keywords, it is necessary to infer the relevance of the words to extract genes and miRNAs in the relevant articles. To address this issue, we use BEST to identify relevant words and genes/miRNAs [21]. BEST has predefined biomedical entities for each category, such as drug, pathway, gene, and disease, and then it identifies relevant entities extracted from PubMed articles from the user query. For example, it returns entities such as ‘ERBB2’, ‘wnt signaling pathway’, and ‘tamoxifen’ with the keyword ‘breast cancer’ as an input. BEST has its own scoring system for entities, which is very useful in ranking gene-miRNA pairs with respect to the user-provided keywords. For example, there are keywords ‘breast cancer’ and entities ‘cell cycle’, ‘mir-200c’, ‘BRCA1’, and ‘ESR’. At the beginning, BEST compiles PubMed articles containing ‘breast cancer’ and the four entities in the abstract. Then, it measures the score and the rank for each entity and lists entities ordered by score. After compiling articles containing ‘BRCA1’ and ‘breast cancer’, BEST calculates a document score for each article and sums the score to measure the entity score, which is denoted as *BEST*(*BreastCancer*, *BRCA*1). In this paper, we use BEST to measure the relevance of each miRNA and mRNA for a given user query.

### Omics score

The **omics score** (OS) is the probability of a gene-miRNA contributing to the class difference when expression data are analyzed. The OS is based on the general principle that differentially expressed miRNA targets genes differentially, resulting in negative correlations between genes and miRNA; then, differentially expressed gene explains the phenotype differences. Context-MMIA computes the **omics score** based on a strategy similar to MMIA. It measures miRNA differential scores, mRNA differential scores, and then correlation scores. The DEmiRNAs and DEmRNAs can be determined by MMIA. After the DEmRNAs and DEmiRNAs are determined, the probability of miRNA-mRNA contributing to the class difference is calculated. Let the p-values of miRNA and mRNA be $p_{m_{i}}$ and $p_{g_{j}}$, respectively. For miRNA *m*_*i*_, *m*_*i*_’s differential score *diff*(*m*_*i*_) is defined by Eq 1, and its normalization *diff*_*n*_(*m*_*i*_) is defined by Eq 2.

$$\text{diff}\left(m_{i}\right)=-log_{2}\left(p_{m_{i}}\right)$$ (1)

$${\text{diff}}_{n}\left(m_{i}\right)=\frac{\text{diff}\left(m_{i}\right)-\min\left(\text{diff}\right)}{\max(\text{diff})-\min(\text{diff})}$$ (2)

The calculation of *diff*_*n*_ for mRNA is similar to that of miRNA. The range of *diff*_*n*_ is between 0 and 1 by Eq 2. If miRNA is significantly differentially expressed in a given condition, then the value of *diff*_*n*_ will be close to 1.

Correlation score is defined by measuring the Pearson’s correlation coefficient of the miRNA-mRNA pair’s logarithmic expression as in [22]. Context-MMIA considers only negatively correlated miRNA-mRNA pairs; thus, a negative value of the coefficient is defined as the correlation score as in Eq 3.

$$corr\left(m_{i},g_{j}\right)=-pearson\_correlation\left(m_{i},g_{j}\right)$$ (3)

The **omics score** of miRNA-mRNA *OS*(*m*_*i*_, *g*_*j*_) is defined in Eq 4.

$$OS\left(m_{i},g_{j}\right)={\text{diff}}_{n}\left(m_{i}\right)*corr\left(m_{i},g_{j}\right)*{\text{diff}}_{n}\left(g_{j}\right)$$ (4)

By definition, *OS*(*m*_*i*_, *g*_*j*_) ∈ [0, 1]; thus, a value of *OS* close to 1 means that the miRNA and mRNA are both significantly differentially expressed and anticorrelated. Thus, we predict that the pair is related to the phenotype difference with a high confidence in terms of expression data.

### Context score

We defined the context score (CS) to measure the probability of a miRNA-mRNA pair contributing to the phenotype difference in terms of the literature information. As described in the previous section, BEST estimates a score between predefined entities and keywords. We denoted the user-input keyword as *k*, which is context specified by the user (e.g., disease, gene, pathway, and so forth). As shown in Eq 5, *CS*(*m*_*i*_, *g*_*j*_|*k*) measures the significance of the *m*_*i*_-*g*_*j*_ pair for *k* in terms of the literature information.

$$CS\left(m_{i},g_{j}|k\right)=P\left(m_{i}|k\right)*P\left(g_{j}|k\right)$$ (5)

To compute *P*(*m*_*i*_|*k*), we used Bayes’ rule and transformed *P*(*m*_*i*_|*k*) into Eq 6 because BEST only measures the score for predefined entities and does not support undefined keywords (e.g., broad keyword, new drug or pathway, and so on) [23].

$$P\left(m_{i}|k\right)=\frac{P_{n}\left(k|m_{i}\right)*P_{n}\left(m_{i}\right)}{\sum_{l=1}^{p}P_{n}\left(k|m_{l}\right)*P_{n}\left(m_{l}\right)}$$ (6)

By converting *P*(*m*_*i*_|*k*) using Bayes’ rule, our method provides the user with a freeform keyword environment, which allows the user to easily utilize our system even when the user is not familiar with biological terms.

$$P\left(k|m_{i}\right)=log_{2}\left(BEST\left(k,m_{i}\right)+1\right)$$ (7)

The literature significance of miRNA (*m*_*i*_) for a given keyword *k*, *P*(*k*|*m*_*i*_), is computed as shown in Eq 7. *BEST*(*k*, *m*_*i*_) is the score of *m*_*i*_ for *k* computed by BEST, and we converted the scale of the score by taking the logarithm of the BEST score. For example, assume that the keyword ‘immune system’ and the miRNA ‘miR-155’ are used in an analysis. If the relation between ‘miR-155’ and ‘immune system’ is well studied, then *P*(*immune* *system* | *miR*155) and *BEST*(*immune system*, *miR*155) will have a high score.

$$P\left(m_{i}\right)=log_{2}\left(BEST\left(m_{i},m_{i}\right)+1\right)$$ (8)

Eq 8 describes how to compute *P*(*m*_*i*_), which denotes how much literature information exists for *m*_*i*_; the more that papers report *m*_*i*_, the higher the value it will have. After computing *P*(*m*_*i*_) and *P*(*k*|*m*_*i*_), normalization terms *P*_*n*_(*m*_*i*_) and *P*_*n*_(*k*|*m*_*i*_) are defined by the min-max normalization.

*P*(*m*_*i*_|*k*) is computed using Bayes’ rule and specifies the significance of *m*_*i*_ given the literature domain *k*, and the value of *P*(*m*_*i*_|*k*) has a correlation with the amount of studies, i.e., the number of papers about *m*_*i*_ in domain *k*. For mRNA *g*_*j*_, *P*(*g*_*j*_|*k*) is computed in a similar way, and we measured the significance of the *m*_*i*_-*g*_*j*_ pair in *k* by computing *CS*(*m*_*i*_, *g*_*j*_|*k*) using *P*(*m*_*i*_|*k*) and *P*(*g*_*j*_|*k*).

### Pair score

The pair score of *m*_*i*_, *g*_*j*_ and *k* is denoted as *Score*(*m*_*i*_, *g*_*j*_, *k*), which is a confidence value of target prediction in terms of both expression and literature data.

$$Score\left(m_{i},g_{j},k\right)=OS\left(m_{i},g_{j}\right)\;*\;CS\left(m_{i},g_{j}\;|\;k\right)$$ (9)

Eq 9 can be interpreted as a weighted **omics score**, where the weight is determined by a probability of a *m*_*i*_, *g*_*j*_ pair being true in terms of the user-provided context given keywords *k*.

## Results

To evaluate Context-MMIA, we performed three experiments in comparison with four existing tools: MMIA, MAGIA2, CoSMic and GenMiR++.

The three experiments were pathway analysis, reproducibility of validated miRNA targets in human, and sensitivity tests when different keywords were used for specifying the experimental context. We used 2-class microarray datasets containing miRNA and mRNA expression profiles in humans. GSE21411 [24], GSE40059 [25], and GSE53482 [26] from human disease studies were used. Each study reports experimentally validated miRNA and the correlated target mRNA pair, which was used to evaluate the miRNA target prediction methods in this section. A detailed description of each dataset is listed in Table 1.

**Table 1:** Dataset summary. Each GEO study comes with an experimentally validated miRNA-mRNA target (the second column) to affect their disease domain (the third column). Disease information was used to test performances when different contexts are specified.

Data	Experimentally validated target	Disease
GSE21411	hsa-miR-23a—NEDD4L	Interstitial Lung Diseases
GSE40059	hsa-miR-200c—CFL2	Breast Cancer
GSE53482	hsa-miR-155—JARID2	Primary Myelofibrosis

Table 1 summarizes the validated target pair and the domain of the experimental design in each dataset. In the interstitial lung diseases (ILD) study, it was reported that ZEB-1 affects the persistence of disease in ILD through suppression of NEDD4L by miR-23a. In the GSE40059 breast cancer study, the authors investigated differences between aggressive breast cancer cell lines and less-aggressive cell lines and reported that CFL2 was up-regulated by miR-200c. The authors also reported that CFL2 expression was correlated with tumor grade. In the primary myelofibrosis (PMF) study, the authors revealed that overexpressed miR-155-5p regulates JARID2, and they suggested that regulated JARID2 may be related to MK hyperplasia in PMF. Disease information was used to test performances when different contexts are specified for Context-MMIA. It is necessary to choose keywords to specify contexts. ‘Interstitial lung disease’ and ‘primary myelofibrosis’ are too specific to use literature data; thus, we used the more general words ‘lung disease’ and ‘myelofibrosis’ as the keywords for Context-MMIA.

### Pathway analysis

To evaluate the effectiveness of the approach used in Context-MMIA, we compared it with four expression-based methods: MMIA, MAGIA2, GenMiR++, and CoSMic. GenMiR++ computes probabilities for target pairs using an EM algorithm. MMIA extracts DEmiRNA to reduce the search space by a user-defined cutoff and finds negatively expressed target DEmRNAs. MAGIA2 provides several methods for the integrated analysis, and we chose Pearson’s correlation method from among these methods. After measuring the correlation, MAGIA2 calculates the false discovery rate (FDR) for each target. CoSMic extracts an mRNA cluster for each miRNA and computes the significance of a cluster using permutation tests. Likewise, each algorithm uses a different strategy to predict the miRNA target and to reduce the search space. We used these four algorithms to compare performances in terms of the predictive power. The methods compute confidence values for the predicted miRNA and mRNA targets, typically probability or p-value. We ranked the prediction results in terms of the confidence values. In the experiments, we used a p-value cutoff of 0.1 for Context-MMIA. For MMIA, a p-value of 0.05 was used for both DEmiRNA and DEmRNA selection.

For the performance evaluation, we used the top 200 predicted miRNA-mRNA pairs predicted by each method. Then, we mapped genes included in the interacting pairs to human pathways using DAVID [27, 28] to determine which pathways were significantly enriched. Among these pathways, we carefully selected pathways that are most likely related to the disease through the literature study as shown in Table 1. We set evaluation criteria as how these literature-guided pathways were predicted by each method. Table 2 shows the ratios of the number of genes that are mapped to significantly enriched pathways to the number of genes included in the top 200 miRNA-target edges. The number of genes is less than 200 because the same gene was multiply targeted, e.g., miR-200c-BRCA1 and miR-23a-BRCA1.

**Table 2:** The ratio of the mapped genes and the number of the genes in the top 200 miRNA-target pairs. From each method, we extracted the top 200 target pairs using each method and performed pathway analysis using DAVID. The numerator is the number of genes mapped to the enriched pathways, and the denominator is the genes in the top 200 edges. The ratio of Context-MMIA is the largest for each dataset.

Methods	GSE21411	GSE40059	GSE53482
Context-MMIA	37 / 79	45 / 157	42 / 127
MMIA	12 / 157	20 / 179	11 / 124
GenMiR++	0 / 194	18 / 197	26 / 200
MAGIA2	18 / 182	12 / 191	19 / 193
CoSMic	24 / 196	9 / 195	X

As shown in Table 2, the number of genes mapped to the significantly enriched pathways is quite different for each method even though the number of genes does not considerably differ for each method. In terms of the ratio of mapped genes to predicted genes, Context-MMIA outperforms the existing methods 2 to 4 times. A gene set in a pathway means that genes have similar biological functions in terms of regulating molecular processes. Thus, the ratios in Table 2 indicate that Context-MMIA produces more functionally coherent gene sets.

Table 3 lists pathways related to ‘breast cancer’ and enriched pathways predicted by each method for the GSE40059 dataset. The enriched pathway analysis for the data from all three experiments is presented in S1 File. The circles in Table 3 mean an enriched pathway when DAVID pathway analysis was performed by using genes in the top 200 edges. For example, if the ECM-receptor interaction is enriched in the Context-MMIA and GenMiR++ results, circles are marked in the context column and the second column for the corresponding tools. As shown in Table 3, more pathways related to ‘breast cancer’ were enriched in the gene sets produced by Context-MMIA than in the gene sets produced by the competing methods. In addition, several important pathways were enriched only in Context-MMIA. For example, it is well known that approximately half of breast tumors have stronger MAP kinase activity than the surrounding benign tissues [32]. Inflammation plays a pivotal role in tumor initiation, promotion, angiogenesis and metastasis. Cytokines are important in all the phenomena, and it has been reported that cytokines participate in regulating both induction and protection in breast cancer [33]. In addition, many studies have reported that TGF-beta signaling is critically important in the regulation of breast cancer [38]. High focal adhesion kinase expression is known to be related to aggressive breast cancer phenotypes [47]. Furthermore, cell adhesion molecules (CAMs) have a strong relationship with the process of metastasis, which is an important feature in predicting breast cancer prognosis [42]. Moreover, a study revealed that activated leukocyte cell adhesion molecule (ALCAM) expression has a correlation with clinical outcomes such as grade, TNM stage, and NPI [48].

**Table 3:** Enriched pathway analysis on GSE40059 breast cancer data. Breast-cancer-related pathways are selected by the literature search. A circle in a cell means that the pathway is enriched by the gene set predicted by each method (A: Context-MMIA, B: MMIA, C: GenMiR++, D: MAGIA2, and E: CoSMic). More pathways are enriched by the gene set in the Context-MMIA result.

Breast-Cancer-Related Pathway	A	B	C	D	E
Purine metabolism [29]		O			
Pyrimidine metabolism [30]		O			
ABC transporters [31]			O		
MAPK signaling pathway [32]	O				
Cytokine-cytokine receptor interaction [33]	O				
Neuroactive ligand-receptor interaction [34]			O		
p53 signaling pathway [35]	O	O			
Apoptosis [36]		O			
Notch signaling pathway [37]				O	
TGF-beta signaling pathway [38]	O				
Axon guidance [39]				O	
Focal adhesion [40]	O	O		O	
ECM-receptor interaction [41]		O			
Cell adhesion molecules (CAMs) [42]	O		O		
Adherens junction [43]	O				
Regulation of actin cytoskeleton [44]	O				
Glioma [45]	O				
Melanoma [46]	O				

### Reproducibility of validated targets in humans

Table 4 shows the rankings of experimentally validated targets among the targets predicted by each method. Because Context-MMIA computes the context score using the literature data for given keywords, there is a possibility that the original papers of the datasets can affect the context score. Thus, we penalized the validated targets to compute *P*(*k*|*m*_*i*_) by excluding each paper when the BEST tool measures a score *BEST*(*k*, *m*_*i*_).

**Table 4:** Reproducibility of validated targets. This table contains the rankings of validated target pairs in three datasets. The validated targets are listed in the second column of Table I. Context-MMIA outperformed existing tools in predicting the validated targets. MAGIA2 and CoSMic failed to reproduce the validated targets.

Data	GSE21411	GSE40059	GSE53482
Context-MMIA	**481**	**338**	**21**
MMIA	1411	387	1465
GenMiR++	8625	1673	95492
MAGIA2	X	X	X
CoSMic	X	X	X (Not Work)

As shown in Table 4, Context-MMIA outperformed the other expression-based methods even though the penalized score is used. MMIA took the second place in reproducing the validated targets, but it ranked validated targets much lower than Context-MMIA. Although not rejecting the validated targets, GenmiR++ ranked validated targets very low. This result shows that GenmiR++ produced too many false positives for the three datasets. MAGIA2 failed to identify the validated targets as positive target pairs in any datasets because none of the validated target pairs satisfied the statistical cutoff. CoSMic also failed to identify the validated target pairs for two datasets, GSE21411 and GSE40059. In addition, CoSMic did not run successfully for dataset GSE53482 due to an input error issue. Many tools were not successful in reproducing validated targets, which can be an indication of false negatives.

To further confirm the reproducibility of our algorithm, we investigated how many experimentally verified targets in humans are detected in the top 200 miRNA-mRNA pairs by each of the methods. Experimentally validated human miRNA-mRNA pairs were extracted from miRTarBase [49], which curated experimentally validated miRNA-target interactions (MTI) by reporter assay, western blot, microarray, and next-generation sequencing experiments. We used human functional MTIs with strong evidence for functionality in humans as true interacting pairs. Table 5 summarizes the number of validated targets in the top 200 miRNA-mRNA pairs predicted by each method.

**Table 5:** Detection of human-specific validated targets. This table contains the number of validated target pairs in three datasets. The validated targets are extracted from miRTarBase target pairs filtered by human functional miRNA target interaction (MTI).

Data	GSE21411	GSE40059	GSE53482
Context-MMIA	**27**	**38**	**24**
MMIA	5	4	12
GenMiR++	3	4	3
MAGIA2	0	0	0
CoSMic	7	0	X (Not Work)

As shown in 5, Context-MMIA predicted two to five times more validated targets compared to the existing methods. Context-MMIA predicted more than 10% of the experimentally validated MTIs in humans, with is a considerably higher prediction accuracy than existing methods; thus, we believe that Context-MMIA suggests good candidates for further experimental validation.

### Sensitivity tests when different keywords are used

The performance of Context-MMIA depends on how the keywords to specify context are related to the goal of the experiment. In addition to disease-related keywords, we performed experiments using less-relevant keywords such as insulin resistance, influenzas, HIV and hepatocellular carcinoma. The results of Context-MMIA using less-relevant keywords are presented in Table 6. The relevant keywords for the three datasets are listed in the third column of Table 1. As shown in Table 6, the rankings of the validated pairs were considerably higher when the keywords that reflect experimental designs were used. This result indicates that our method is able to reflect the degree of relevance to the experimental design and capture the different miRNA-mRNA pairs when different keywords were used. In summary, the experiments with irrelevant keywords showed that our method can capture the miRNA-mRNA pairs, reflecting the user-specified biological context.

**Table 6:** Sensitivity tests when different keywords are used. Rankings of validated targets are shown when different keywords are used. The validated targets had high ranks when disease-related keywords were used.

Keyword	GSE21411	GSE40059	GSE53482
Correct keyword	**481**	**338**	**21**
Insulin resistance	12479	2036	4250
Influenzas	6826	1169	1623
HIV	5865	4002	3238
Hepatocellular carcinoma	5278	3265	7180

## Conclusion

We presented Context-MMIA, a human-specific miRNA-mRNA target pair prediction system that utilizes both expression profiles and the literature information from the user-specified experimental design goals. A major contribution of our system is that we handled the false positives and false negatives, which are an inherent issue in expression-based prediction tools, by incorporating the user-specified context information from the literature. Analyses on three independent human datasets showed that Context-MMIA can capture the true positive miRNA-mRNA target pairs that are specific to a biological context. Context-MMIA outperformed existing tools in a series of experiments, such as pathway analysis, validated target ranking, and irrelevant keyword experiments.

We emphasize that computational predictions of miRNA-mRNA target pairs should be further validated in biological experiments and that our system is intended to provide good candidates for experimental validation. Context-MMIA is available at http://biohealth.snu.ac.kr/software/contextMMIA

## Supporting information

S1 File Pathway analysis results. S1 File contains pathway results for the other two datasets.
(PDF)
