# Supplementary material for: Literature-based condition-specific miRNA-mRNA target prediction
Source: PLoS One. 2017 Mar 31;12(3):e0174999. doi: 10.1371/journal.pone.0174999 (PMC5376335; doi:10.1371/journal.pone.0174999)
Supplement: S1 File — contains pathway results for the other two datasets. (PDF) [file pone.0174999.s001.pdf]

TABLE I: Enriched pathway analysis on GSE21411 interstitial lung disease. ILD related pathways are selected by the literature search. Circle in cell means that the pathway is enriched by the gene set predicted by each method.

	Context-MMIA	MMIA	GenMir++	MAGIA2	COSMIC
Glycine serine and threonine metabolism [1]	O				
MAPK signaling pathway [2]	O				
ErbB signaling pathway [3]	O				
Cytokine-cytokine receptor interaction [4]	O				
Chemokine signaling pathway [5]	O				
p53 signaling pathway [6]	O				
Endocytosis [7]	O				
mTOR signaling pathway [8]		O			
Apoptosis [9]	O				
VEGF signaling pathway [10]	O				
Focal adhesion [11]	O				O
ECM-receptor interaction [11]					O
Cell adhesion molecules (CAMs) [12]					
Adherens junction [13]					
Toll-like receptor signaling pathway [14]	O				
T cell receptor signaling pathway [15]	O	O			
B cell receptor signaling pathway [1]	O				
Insulin signaling pathway [1]		O			
Melanoma [16]					O

TABLE II: Enriched pathway analysis on GSE53482 primary myelofibrosis. PMF related pathways are selected by the literature search. Circle in cell means that the pathway is enriched by the gene set predicted by each method.

	Context-MMIA	MMIA	GenMir++	MAGIA2	COSMIC
MAPK signaling pathway [17]		O		O	
Calcium signaling pathway [18]			O		
Cytokine-cytokine receptor interaction [19]	O				
Chemokine signaling pathway [18]	O				
Neuroactive ligand-receptor interaction [20]			O		
Cell cycle[21]				O	
Lysosome [22]		O			
Endocytosis [23]	O				
TGF-beta signaling pathway [24]	O				
Focal adhesion [25]	O				
ECM-receptor interaction [25]	O		O		
Regulation of actin cytoskeleton [18]	O				
Chronic myeloid leukemia [26]	O			O	
Acute myeloid leukemia [26]	O				

Table I and Table II list each disease related pathways and enriched pathways predicted by each method for GSE21411 and GSE53482 datasets. Similar with GSE40059 result, it were enriched in gene sets produced by Context-MMIA than the gene sets produced by the competing methods.

In ILD, it is reported that MAPK receptor signaling pathway is core regulator of oncogenic transformation and tumor maintenance [27]. It is found that MAPK pathway mediates the proliferative response of lung fibroblasts [28]. Also there is the study in healthy African American(AA) skin and lung tissue found that chemokine receptors are upregulated in healthy AA and suggested that chemokine receptors contribute progression of skin and lung fibrosis [29]. Idiopathic pulmonary fibrosis (IPF) is the most iethal ILD, it is reported that there is increased epithelial apoptosis in lungs of IPF patients [9]. The TOLLIP have a negative interaction with Toll-like receptor signaling, which variants have a relation to survival in IPF [15].

In PMF, endocytosis regulates TGF-beta level, which is critical component of myelofibrosis development [23]. It is reported that increasing TGF-beta signaling is associated with PMF and it may represent targets for therapeutic intervention of PMF [24]. Also a study suggested that multiple alterations of focal adhesion and ECM in bone marrow could contribute TGF-beta activation in PMF patients [25].

Reference

[1] J.-H. Cho, R. Gelinas, K. Wang, A. Etheridge, M. G. Piper, K. Batte, D. Dakhlallah, J. Price, D. Bornman, S. Zhang et al., "Systems biology of interstitial lung diseases: integration of mrna and microrna expression changes," BMC medical genomics, vol. 4, no. 1, p. 1, 2011.
[2] K. M. Antoniou, G. A. Margaritopoulos, G. Soufla, E. Symvoulakis, E. Vassalou, R. Lymbouridou, K. D. Samara, D. Kappou, D. A. Spandidos, and N. M. Siafakas, "Expression analysis of akt and mapk signaling pathways in lung tissue of patients with idiopathic pulmonary fibrosis (ipf)," Journal of Receptors and Signal Transduction, vol. 30, no. 4, pp. 262–269, 2010.
[3] C. Li, R. Wei, Y. L. Jones-Hall, R. Vittal, M. Zhang, and W. Liu, "Epidermal growth factor receptor (egfr) pathway genes and interstitial lung disease: an association study," Scientific reports, vol. 4, p. 4893, 2014.
[4] B. D. Bringardner, C. P. Baran, T. D. Eubank, and C. B. Marsh, "The role of inflammation in the pathogenesis of idiopathic pulmonary fibrosis," Antioxidants & redox signaling, vol. 10, no. 2, pp. 287–302, 2008.
[5] I. Sabroe, C. Lloyd, M. Whyte, S. Dower, T. Williams, and J. Pease, "Chemokines, innate and adaptive immunity, and respiratory disease," European Respiratory Journal, vol. 19, no. 2, pp. 350–355, 2002.
[6] K. Kuwano, R. Kunitake, M. Kawasaki, Y. Nomoto, N. Hagimoto, Y. Nakanishi, and N. Hara, "P21waf1/cip1/sdi1 and p53 expression in association with dna strand breaks in idiopathic pulmonary fibrosis." American journal of respiratory and critical care medicine, vol. 154, no. 2, pp. 477–483, 1996.
[7] S. Cappelli, S. B. Randone, G. Camiciottoli, A. De Paulis, S. Guiducci, and M.Matucci-Cerinic, "Interstitial lung disease in systemic sclerosis: where do we stand?" European Respiratory Review, vol. 24, no. 137, pp. 411–419, 2015.
[8] E. A. Goncharova, "mtor and vascular remodeling in lung diseases: current challenges and therapeutic prospects," The FASEB Journal, vol. 27, no. 5, pp. 1796–1807, 2013.
[9] B. Uhal, "The role of apoptosis in pulmonary fibrosis," European Respiratory Review, vol. 17, no. 109, pp. 138–144, 2008.
[10] M. M. Wieck, R. G. Spurrier, D. E. Levin, S. G. Mojica, M. J. Hiatt, R. Reddy, X. Hou, S. Navarro, J. Lee, A. Lundin et al., "Sequestration of vascular endothelial growth factor (vegf) induces late restrictive lung disease," PloS one, vol. 11, no. 2, p. e0148323, 2016.
[11] G. Bagnato and S. Harari, "Cellularinteractions in the pathogenesis of interstitial lung diseases," European Respiratory Review, vol. 24, no. 135, pp. 102–114, 2015.
[12] A. Southcott, I. Hemingway, S. Lorimer, K. Sugars, P. Hellewell, C. Black, P. Jeffery, A. Gearing, D. Haskard, and R. Du Bois, "Adhesion molecule expression in the lung: a comparison between normal and diffuse interstitial lung disease," European Respiratory Journal, vol. 11, no. 1, pp. 91–98, 1998.
[13] H. M. Karvonen, S. T. Lehtonen, R. T. Sormunen, T. H. Harju, E. Lappi-Blanco, R. S. Bloigu, and R. L. Kaarteenaho, "Myofibroblasts in interstitial lung diseases show diverse electron microscopic and invasive features," Laboratory Investigation, vol. 92, no. 9, pp. 1270–1284, 2012.
[14] M. A. Kovach and T. J. Standiford, "Toll like receptors in diseases of the lung," International immunopharmacology, vol. 11, no. 10, pp. 1399–1406, 2011.
[15] A. Tochimoto, Y. Kawaguchi, and H. Yamanaka, "Genetic susceptibility to interstitial lung disease associated with systemic sclerosis,"Clinical medicine insights. Circulatory, respiratory and pulmonary medicine, vol. 9, no. Suppl 1, p. 135, 2015.
[16] S. Ikeda, M. Arita, M. Morita, S. Ikeo, A. Ito, F. Tokioka, M. Noyama, K. Misaki, K. Notohara, and T. Ishida, "Interstitial lung disease in clinically amyopathic dermatomyositis with and without anti-mda-5 antibody: to lump or split?" BMC pulmonary medicine, vol. 15, no. 1, p. 1, 2015.
[17] C. Desterke, C. Martinaud, B. Guerton, L. Pieri, C. Bogani, D. Clay, F. Torossian, J.-J. Lataillade, H. C. Hasselbach, H. Gisslinger et al., "Tetraspanin cd9 participates in dysmegakaryopoiesis and stromal interactions in primary myelofibrosis," haematologica, vol. 100, no. 6, pp. 757–767, 2015.
[18] E. Calura, S. Pizzini, A. Bisognin, A. Coppe, G. Sales, E. Gaffo, T. Fanelli, C. Mannarelli, R. Zini, R. Norfo et al., "A data-driven network model of primary myelofibrosis: transcriptional and post-transcriptional alterations in cd34+ cells," Blood Cancer Journal, vol. 6, no. 6, p. e439, 2016.
[19] O. I. Abdel-Wahab and R. L. Levine, "Primary myelofibrosis: update on definition, pathogenesis, and

treatment," Annual review of medicine, vol. 60, pp. 233–245, 2009.
[20] P. Guglielmelli, C. Bogani, N. Bartalucci, L. Pieri, A. Pancrazzi, A. Bosi, A. Bisognin, M. Aracil, S. Bortoluzzi, and A. M. Vannucchi,"Characterization of targets of plitidepsin in jak2v617f-mutated cells from myeloproliferative neoplasms," Blood, vol. 116, no. 21, pp. 4093–4093, 2010.
[21] T. Kumagai, A. Tefferi, L. Jones, and H. P. Koeffler,"Methylation analysis of the cell cycle control genes in myelofibrosis with myeloid metaplasia," Leukemia research, vol. 29, no. 5, pp. 511–515, 2005.
[22] A. Tabarroki and R. V. Tiu, "Molecular genetics of myelofibrosis and its associated disease phenotypes," Translational medicine@ UniSa, vol. 8, p. 53, 2014.
[23] M. Bender, S. Giannini, R. Grozovsky, T. J¨onsson, H. Christensen, F. G. Pluthero, A. Ko, A. Mullally, W. H. Kahr, K. M. Hoffmeister et al., "Dynamin 2–dependent endocytosis is required for normal megakaryocyte development in mice," Blood, vol. 125, no. 6, pp. 1014–1024, 2015.
[24] M. Zingariello, F. Martelli, F. Ciaffoni, F. Masiello, B. Ghinassi, E. DAmore, M. Massa, G. Barosi, L. Sancillo, X. Li et al., "Characterization of the tgf-1 signaling abnormalities in the gata1low mouse model of myelofibrosis," Blood, vol. 121, no. 17, pp. 3345–3363, 2013.
[25] C. Desterke, C. Martinaud, N. Ruzehaji, and M.-C. Le Bousse-Kerdil`es,"Inflammation as a keystone of bone marrow stroma alterations in primary myelofibrosis," Mediators of inflammation, vol. 2015, 2015.
[26] A. Tefferi, "Primary myelofibrosis: 2013 update on diagnosis, riskstratification, and management," American journal of hematology, vol. 88, no. 2, pp. 141–150, 2013.
[27] M. L. Sos, S. Fischer, R. Ullrich, M. Peifer, J. M. Heuckmann, M. Koker, S. Heynck, I. St¨uckrath, J.Weiss, F. Fischer et al., "Identifying genotypedependent efficacy of single and combined pi3k-and mapk-pathway inhibition in cancer," Proceedings of the National Academy of Sciences, vol. 106, no. 43, pp. 18 351–18 356, 2009.
[28] S. Matthiesen, A. Bahulayan, O. Holz, and K. Rack´e,"Mapk pathway mediates muscarinic
